# Association of the Hermansky-Pudlak syndrome type-3 protein with clathrin

**DOI:** 10.1186/1471-2121-6-33

**Published:** 2005-09-13

**Authors:** Amanda Helip-Wooley, Wendy Westbroek, Heidi Dorward, Mieke Mommaas, Raymond E Boissy, William A Gahl, Marjan Huizing

**Affiliations:** 1 Section on Human Biochemical Genetics, Medical Genetics Branch, National Human Genome Research Institute, NIH, Bethesda MD, USA; 2 Department of Molecular Cell Biology, Leiden University Medical Center, Leiden, Netherlands; 3 Department of Dermatology, University of Cincinnati College of Medicine, OH, USA

## Abstract

**Background:**

Hermansky-Pudlak syndrome (HPS) is a disorder of lysosome-related organelle biogenesis characterized by oculocutaneous albinism and prolonged bleeding. These clinical findings reflect defects in the formation of melanosomes in melanocytes and dense bodies in platelets. HPS type-3 (HPS-3) results from mutations in the *HPS3 *gene, which encodes a 1004 amino acid protein of unknown function that contains a predicted clathrin-binding motif (LLDFE) at residues 172–176.

**Results:**

Clathrin was co-immunoprecipitated by HPS3 antibodies from normal but not HPS3 null melanocytes. Normal melanocytes expressing a GFP-HPS3 fusion protein demonstrated partial co-localization of GFP-HPS3 with clathrin following a 20°C temperature block. GFP-HPS3 in which the predicted clathrin-binding domain of HPS3 was mutated (GFP-HPS3-delCBD) did not co-localize with clathrin under the same conditions. Immunoelectron microscopy of normal melanocytes expressing GFP-HPS3 showed co-localization of GFP-HPS3 with clathrin, predominantly on small vesicles in the perinuclear region. In contrast, GFP-HPS3-delCBD did not co-localize with clathrin and exhibited a largely cytoplasmic distribution.

**Conclusion:**

HPS3 associates with clathrin, predominantly on small clathrin-containing vesicles in the perinuclear region. This association most likely occurs directly via a functional clathrin-binding domain in HPS3. These results suggest a role for HPS3 and its protein complex, BLOC-2, in vesicle formation and trafficking.

## Background

Hermansky-Pudlak syndrome (HPS [MIM: 203300]) is an autosomal recessive disorder of vesicle biogenesis resulting in the dysfunction of lysosome-related organelles such as melanosomes and platelet dense bodies [1-4]. Affected patients have oculocutaneous albinism presenting as congenital nystagmus, reduced visual acuity, and varying degrees of hypopigmentation of the skin, hair, and irides [5-7]. In addition, a platelet storage pool deficiency, manifesting as absence of platelet dense bodies, causes loss of the secondary aggregation response [2,8,9]. Clinically, this results in easy bruising and epistaxis in childhood, prolonged bleeding during dental extractions and surgeries, and excessive menstrual and postpartum bleeding [9]. Some HPS patients also develop granulomatous colitis or a fatal pulmonary fibrosis [9,10].

To date, seven genes have been identified as causes of human HPS subtypes (HPS-1 through HPS-7), and other genes identified in mouse models of HPS may also cause HPS in humans [4,11]. Of the human subtypes, only HPS-2 ([MIM: 603401]) results from mutations in a gene (*AP3B1*) with a known function. *AP3B1 *codes for the β3A subunit of adaptor complex-3 (AP-3), a coat protein that is involved in sorting transmembrane proteins to lysosomes and lysosome-related organelles [12-15]. This recognized function of AP-3 supports the paradigm that all types of HPS result from abnormal vesicle formation and/or trafficking.

Each of the gene products of *HPS1 *([MIM: 604982; [16,17]]), *HPS3 *([MIM: 606118; [18,19]]), *HPS4 *([MIM: 606682; [20,21]]), *HPS5 *([MIM: 607521; [22,23]]), *HPS6 *([MIM: 607522; [22]]), and *HPS7 *([MIM: 607145; [24]]) is unique, although some HPS proteins interact with each other in Biogenesis of Lysosome-related Organelles Complexes or BLOCs [22,24-26]. The fact that these proteins have no homology to any known proteins, to each other, or to known functional domains makes them challenging candidates to investigate using *in vitro *methods.

In an attempt to understand the function of HPS gene products, we focused on HPS3, a unique protein with a predicted clathrin-binding domain. HPS-3 patients, with mutations in the *HPS3 *gene, have absent platelet dense bodies, mild to moderate hypopigmentation of skin and hair, iris transillumination, and patchy hypopigmentation of the fundus [19]. The mouse ortholog of human HPS-3, *cocoa*, demonstrates similar features [27,28]. The *HPS3 *gene was identified by homozygosity mapping using HPS patients from a central Puerto Rican genetic isolate [18]. *HPS3 *is located on chromosome 3q24 and has 17 exons and a 3,015-bp open reading frame that codes for a 1004-amino acid protein. The central Puerto Rican founder mutation in *HPS3 *is a 3.9-kb deletion encompassing exon 1 and its surrounding introns [18]. The central Puerto Rican HPS population is distinct from the HPS isolate in northwest Puerto Rico, in which a founder mutation in the *HPS1 *gene results in a severe form of HPS [16]. Several non-Puerto Rican *HPS3 *mutations, as well as an Ashkenazi Jewish founder mutation in *HPS3*, have now been identified [19].

The human HPS3 protein contains a predicted clathrin-binding motif (LLDFE) at residues 172–176 that conforms to the consensus sequence L(L, I)(D, E, N)(L, F)(D, E). This consensus was determined by amino acid sequence alignment of the clathrin-binding regions in the beta subunits of adaptor proteins 1, 2, and 3 (β1, β2, β3A, and β3B), arrestin 3, and amphiphysin I and II [29]. This 'clathrin box' is sufficient for binding to the amino terminal domain of the clathrin heavy chain [29]. The structures of peptide complexes containing the clathrin-terminal domain and the clathrin-binding motifs of β-arrestin 2 and β3A have been determined by crystallography [30]. Both of these peptides bind, via their clathrin box motifs, to the same site in the clathrin heavy chain's terminal domain, with nearly identical bound conformations.

Clathrin is the main component of protein coats that assist in the formation of vesicles budding from the trans-Golgi network (TGN), plasma membrane, and endosomes. Several clathrin-associated proteins regulate the assembly of clathrin triskelions, composed of three heavy chains and three light chains, into the polyhedral cages that provide structure to intracellular vesicles. Some of these proteins and protein complexes are also involved in sorting cargo into vesicles and directing their transport within the cell [31,32].

HPS-2 disease results from deficiency of the clathrin binding protein β3A [12-15], supporting a possible role for other HPS proteins in clathrin binding. Hence, we investigated the clathrin binding function of the HPS3 protein in melanocytes and fibroblasts.

## Results

Several avenues of investigation were pursued to substantiate the predicted clathrin-binding activity of HPS3. These studies employed the techniques of immunoprecipitation, immunofluorescence, live cell imaging, and immunoelectron microscopy.

### Immunoprecipitation

**Figure 1:**
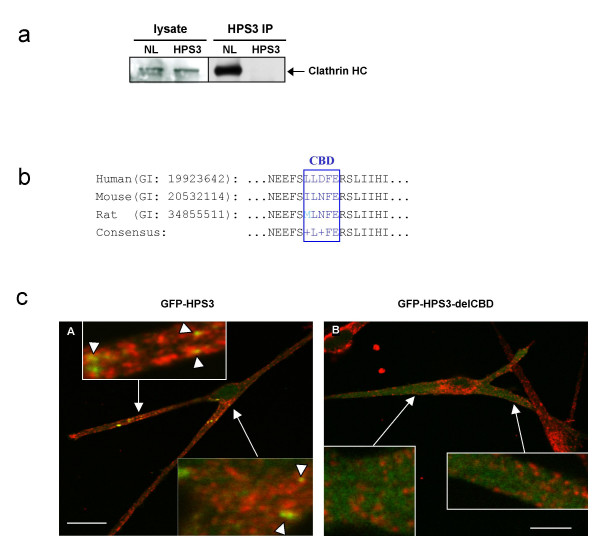
**Association of HPS3 with clathrin**. (a) Immunoprecipitation of clathrin with HPS3 antibodies. Western blots of normal (NL) and HPS3-null (HPS3) melanocyte lysates treated with clathrin heavy chain antibodies. Equal amounts of clathrin were detected in both lysates [left panel]. The lysates were immunoprecipitated with HPS3 antibodies, electrophoresed, and immunoblotted using clathrin heavy chain antibodies. HPS3 antibodies immunoprecipitated clathrin only in the normal and not in the HPS3-null melanocyte lysates [right panel]. (b) Amino acid sequences of the predicted clathrin-binding domain (residues 172–176 of human HPS3, shown in blue) in the human, mouse and rat HPS3 proteins, and the surrounding amino acid sequences. (c) Confocal immunofluorescence microscopy of representative normal melanocytes electroporated with GFP-HPS3 [A] and GFP-HPS3-delCBD [B] (in green) and co-stained with antibodies to clathrin (in red). GFP-HPS3 partially co-localized with clathrin on small vesicles (arrowheads) [A], but GFP-HPS3-delCBD did not [B]. (Bar = 20 μm).

Whole cell lysates prepared from normal melanocytes and from HPS3 null melanocytes, i.e., cells from a patient homozygous for the 3.9-kb *HPS3 *founder deletion [18], exhibited approximately equal amounts of clathrin (Figure 1a, left panel). Each whole cell lysate was then immunoprecipitated with polyclonal HPS3 peptide antibodies. Clathrin, detected by antibodies to its heavy chain, co-immunoprecipitated with HPS3 in the normal lysates but not in the HPS3 null lysates (Figure 1a, right panel). These results were obtained using either of two different monoclonal anti-clathrin heavy chain antibodies.

### Immunofluorescence

At residues 172–176, the HPS3 protein has a predicted clathrin-binding motif (LLDFE) that is conserved in mouse and rat (Figure 1b). Using site-directed mutagenesis, a GFP-HPS3-delCBD construct was created in which the clathrin-binding motif was converted to non-conserved amino acids (AAAPG). The interaction of clathrin-binding proteins with clathrin is a transient phenomena [31,33,34]. To maximize the likelihood of observing the association of GFP-HPS3 with clathrin by immunofluorescence, a 20°C temperature block was employed. Incubation at 20°C for 2 h was used to block trafficking out of the trans-Golgi [35,36] and followed by transfer to 37°C for 5 min to release the temperature block and resume normal trafficking. Normal melanocytes expressing wild-type GFP-HPS3 or GFP-HPS3-delCBD, so treated, were then fixed and stained with clathrin heavy chain antibodies. Cells expressing GFP-HPS3 demonstrated partial co-localization with clathrin on one to several small vesicles in the perinuclear area (representative cell, Figure 1c[A]). In a representative experiment, 9 of 11 GFP-HPS3 expressing melanocytes showed this co-localization. In contrast, 0 of 10 cells exhibited co-localization of GFP-HPS3-delCBD with clathrin (Figure 1c[B]; p < 0.001 by Chi-square analysis).

### Live cell imaging

**Figure 2:**
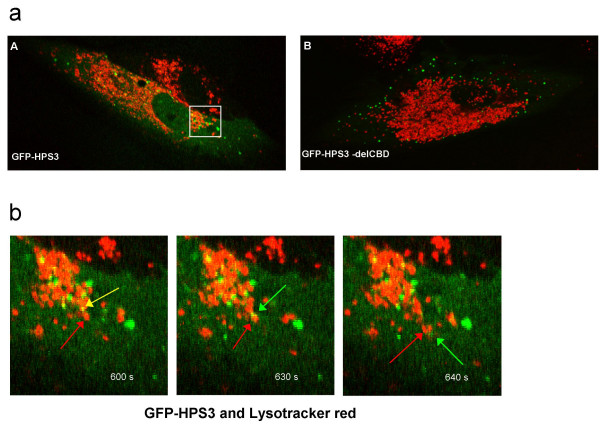
**Live cell imaging of HPS3 and acidic vesicles**. (a) Lysotracker Red stained acidic vesicle trafficking in live HPS3 null fibroblasts expressing GFP-HPS3 [A] or GFP-HPS3-delCBD [B]. Cells were imaged on a 37°C warming stage following a 2 h incubation at 20°C. Lysotracker Red stained acidic vesicles and GFP-HPS3 transiently co-localized in the perinuclear area [A]. Inset shows the location of the time series shown in (b). GFP-HPS3-delCBD did not localize predominantly to the perinuclear area and showed no co-localization with Lysotracker Red [B]. (b) Time series showing co-trafficking of GFP-HPS3 and Lysotracker Red stained vesicles from the perinuclear area in HPS3 null fibroblasts. Note transient association of GFP-HPS3 with an acidic vesicle as it travels peripherally over a period of time (see [additional file 1]).

Trafficking of acidic vesicles, stained with Lysotracker Red, was followed in live HPS3 null fibroblasts expressing either GFP-HPS3 (Figure 2a[A]) or GFP-HPS3-delCBD (Figure 2a[B]). Following a 20°C temperature block and transfer to 37°C, acidic vesicles and GFP-HPS3 clustered together in the perinuclear area (Figure 2b[A]). GFP-HPS3 transiently interacted with acidic vesicles and, in some cases (arrows, Figure 2b), emerged together with them from the perinuclear area (Figure 2b and [Additional file 1]). In contrast, following a 20°C block and transfer to 37°C, GFP-HPS3-delCBD was predominantly localized in a punctate pattern in the periphery of the cell; no association of GFP-HPS3-delCBD with acidic vesicles was observed (Figure 2a[B]).

### Immunoelectron microscopy

Normal melanocytes were fixed approximately 9 h after transfection with GFP-HPS3. Expression of the GFP-HPS3 fusion protein was low at this time, thus minimizing aggregation and other artifacts that can occasionally be observed in overexpressing cells. Normal melanocytes transfected with GFP-HPS3-delCBD were fixed later (approximately 24 h after transfection) because of the very low levels of expression obtained with this construct. No aggregates were observed in the GFP-HPS3-delCBD transfected cells.

**Figure 3:**
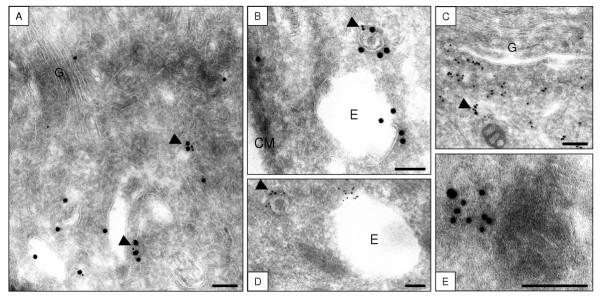
**Immunoelectron micrographs demonstrating co-localization of GFP-HPS3 and clathrin**. Double-labeling of anti-GFP (10-nm gold) and anti-clathrin (20-nm gold) [A, B], or the reverse labeled anti-clathrin (10-nm gold) and anti-GFP (15-nm gold) [C, D, E] in normal melanocytes electroporated with GFP-HPS3. Co-localization of the two labels was shown on small (50–100 nm) clathrin-containing vesicles (arrowheads) in the Golgi region [A and C]. Co-localization was observed on small clathrin-labeled vesicles (arrowheads) but not on neighboring large endosomal structures [B and D]. High magnification of a small clathrin containing vesicle labeled with GFP-HPS3 [E]. CM = Cell Membrane, G = Golgi area, E = Endosomal structure. Bar = 100 nm

GFP-HPS3 localized predominantly to small (50–100 nm) vesicles in the Golgi region (57 of 111 (51%) anti-GFP immunogold particles; Table 1) and the vast majority of these particles (53 of 57 (93%)) co-localized with clathrin (Figure 3, arrowheads; Table 1). At high magnification, GFP-HPS3 labeling was demonstrated on well-defined clathrin-containing vesicles (Figure 3[B,D,E]). Some of these small clathrin-containing vesicles were found near larger endosomal structures (Figure 3[B,D]). GFP-HPS3 labeling was generally less abundant on large endosomal structures than on small vesicles (23 of 111 (21%) of anti-GFP immunogold particles found on endosomes) and fewer of these particles co-localized with clathrin (15 of 23 (65%); Figure 3[B,D], Table 1).

**Table 1:** Quantitation of immunogold label in normal melanocytes transfected with GFP-HPS3 or GFP-HPS3-delCBD^1^

	**Gold Particles**		**Clathrin Association**	
**Intracellular compartment**	**GFP-HPS3**	**GFP-HPS3-delCBD**	**GFP-HPS3**	**GFP-HPS3-delCBD**

**Small vesicles in Golgi area ^2,3^**	57	8	53	1
**Golgi stacks**	1	1	0	0
**Other small vesicles**	15	16	9	3
**Endosomal structures ^3^**	23	17	15	1
**Mitochondria**	2	2	0	0
**Melanosomes**	3	2	0	0
**Nucleus**	2	1	0	0
**Cytoplasm ^2^**	8	63	0	3
				
**Total **^3^	111	110	77	8

^1 ^Anti-GFP immunogold particles were counted in a total of 28 GFP-HPS3 and 25 GFP-HPS3-delCBD expressing cells

^2 ^Difference in number of gold particles per compartment, for GFP-HPS3-delCBD compared with GFP-HPS3, is statistically significant by Chi square analysis (p < 0.001)

^3 ^Difference in number of gold particles found in association with clathrin, for GFP-HPS3-delCBD compared with GFP-HPS3, is statistically significant by Chi square analysis (p < 0.001)

**Figure 4:**
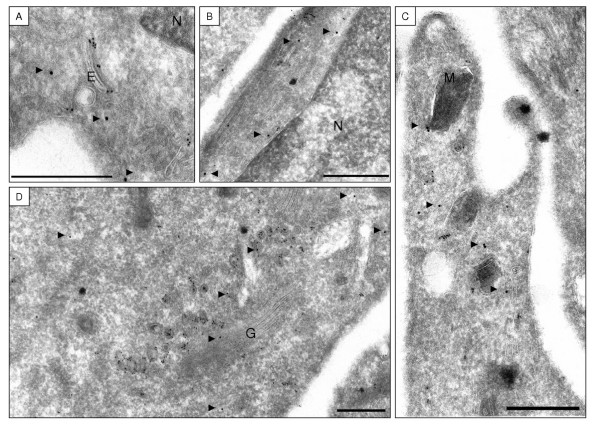
**Immunoelectron micrographs demonstrating no co-localization of GFP-HPS3-delCBD and clathrin.** Double-labeling of anti-clathrin (10-nm gold) and anti-GFP (15-nm gold) in normal  melanocytes electroporated with GFP-HPS3-delCBD [A-D].  GFP-HPS3-delCBD (arrowheads) was largely cytoplasmic and distributed throughout the entire cell, from the perinuclear/Golgi region [A,B,D] to the tips [C].  No co-localization of the two labels (GFP-HPS3-delCBD and clathrin) was observed.  N=Nucleus, G=Golgi, E=Endosomal structure, M=Melanosome.  Bar = 500 nm

In contrast, GFP-HPS3-delCBD expressing cells demonstrated very few anti-GFP immunogold particles on small vesicles in the Golgi region (8 of 110 (7%)) and these were only rarely found in association with clathrin (1 of 8 (13%); Figure 4, Table 1). The majority of anti-GFP immunogold particles localized to the cytoplasm of GFP-HPS3-delCBD expressing cells (63 of 110 (57%)), compared with only 7% (8 of 111) in GFP-HPS3 expressing cells (Figures 3 and 4, Table 1). These differences were statistically significant by Chi square analysis (p < 0.001; Table 1).

No appreciable GFP-HPS3 or GFP-HPS3-delCBD labeling was observed on clearly identifiable Golgi stacks (Figure 3[A,C], Figure 4[D], Table 1). As expected, clathrin labeling appeared much more abundant than GFP labeling in GFP-HPS3 or GFP-HPS3-delCBD expressing melanocytes. Similar amounts of GFP labeling per cell were detected in GFP-HPS3 and GFP-HPS3-delCBD expressing cells and no GFP labeling was observed in untransfected cells. Not all clathrin-containing membranes were associated with GFP-HPS3, but the majority of GFP-HPS3 appeared to be associated with a clathrin-containing membrane.

## Discussion

HPS3 is unusual among HPS proteins in that it contains a predicted functional domain, i.e., a clathrin-binding motif (LLDFE) (Figure 1b). In this report, we describe evidence for the association of HPS3 with clathrin. We demonstrated that clathrin co-immunoprecipitates with endogenous HPS3 in normal melanocytes; no clathrin was immunoprecipitated in the absence of HPS3, i.e., in HPS3 null cells. The necessity of the clathrin-binding domain in HPS3 is supported by the partial co-localization of GFP-HPS3 with clathrin only when this domain is present, as demonstrated by both fluorescence and immunoelectron microscopy studies. Furthermore, localization and trafficking of GFP-HPS3 with acidic vesicles (labeled with Lysotracker Red) depends upon an intact clathrin-binding domain.

The presence of a conserved clathrin-binding motif in HPS3, combined with the supportive data mentioned above, indicates that HPS3 most likely binds clathrin directly. We cannot, however, rule out the possibility that HPS3 interacts indirectly with clathrin. Recent studies in mouse [37] and human [38] have shown that HPS3 (*cocoa *mouse) interacts with HPS5 (*ruby-eye-2 *mouse) and HPS6 (*ruby-eye *mouse) in the BLOC-2 complex. Hence, HPS3 may function as an essential component of a complex, such as BLOC-2, in which another member binds clathrin. This seems unlikely, however, since HPS5 and HPS6 have no conserved clathrin-binding sequence motifs.

In our ultrastructural studies, GFP-HPS3 was found primarily on small (50 to 100 nm) clathrin containing vesicles in the perinuclear/Golgi region of normal melanocytes. Mutation of the clathrin-binding domain of HPS3 resulted in a largely cytoplasmic distribution of the fusion protein, suggesting that the clathrin-binding domain is necessary for the correct localization of HPS3 and that clathrin recruits HPS3 to small vesicles. Interestingly, no GFP-HPS3 was seen on Golgi stacks or on vesicles budding from the TGN, nor was it localized to large endosomal structures or mature melanosomes. In melanocytes from HPS3 deficient patients, DOPA histochemistry (used to identify extra-melanosomal sites of functional tyrosinase) detected 50 nm DOPA positive vesicles distributed throughout the cell [39]. This was in contrast to the situation in normal melanocytes, in which the small DOPA-positive vesicles were restricted to the Golgi region. A possible explanation is that HPS3 (and perhaps BLOC-2 as a whole) interacts with the small DOPA-positive vesicles via its clathrin-binding domain, escorting them from the Golgi region to premelanosomes for delivery of their contents. In such a scenario, additional specialized accessory factors (perhaps other HPS proteins or BLOCs) would regulate vesicle targeting, budding and fusion events, clathrin coat assembly and disassembly, and interactions with the cytoskeleton.

Future investigations should pursue the function of BLOC-2 with the recognition that one of its components, HPS3, binds clathrin and may, therefore, bind to vesicles. This understanding allows for hypotheses regarding the roles of HPS5 and HPS6 in BLOC-2. Possible roles could include such functions as binding designated cargo, regulating conformational changes of the complex, or tethering proteins or vesicles for interactions with the BLOC-2 complex as a whole.

## Conclusion

HPS3 associates with clathrin, predominantly on small clathrin-containing vesicles in the perinuclear region. This association most likely occurs directly via a functional clathrin-binding domain in HPS3 and is supported by immunoprecipitation, immunofluorescence, live cell imaging and immunoelectron microscopy data.

## Methods

### Patients and cells

Normal human primary epidermal melanocytes used for immunoelectron microscopy were obtained from neonatal foreskin and established as described [40,41]. HPS-3 patient (HPS3 null) primary epidermal melanocytes and primary fibroblast cultures were obtained from skin biopsies and cultured as previously described [14]. All other normal human primary epidermal melanocytes were purchased from Cascade Biologics (Portland, OR). The HPS-3 patients were enrolled in a protocol approved by the National Human Genome Research Institute Institutional Review Board to study the clinical and molecular aspects of HPS. Written informed consent was obtained from the patient or the patient's parent.

### Immunoprecipitation and western blotting

Normal and HPS3 null melanocytes cell pellets were resuspended in PBS containing 1% NP-40 and protease inhibitors (Roche Molecular Biochemicals, Indianapolis, IN). The cell lysates were cleared by centrifugation and the resulting supernatants were incubated with HPS3 polyclonal peptide antibody (developed in rabbit against the peptide KMGDLDMHRNEMKSHS) followed by incubation with protein A/G agarose beads (Oncogene Research Products, San Diego, CA). The agarose beads were washed, boiled in SDS-loading buffer and centrifuged. The resulting supernatants were electrophoresed on 4–12% SDS-PAGE gels (Invitrogen, Carlsbad, CA) and electro-blotted onto nitrocellulose membranes (Schleicher and Schuell, Keene, NH). The membranes were blocked and incubated with mouse monoclonal anti-clathrin antibodies (1:1000) (BD Biosciences Pharmingen (San Diego, CA) or Affinity BioReagents (Golden, CO), followed by incubation with HRP-conjugated anti-mouse IgG secondary antibodies (1:3000; Amersham Biosciences, Piscataway, NJ). Results were visualized with enhanced chemiluminescence (ECL Western Blotting Detection Reagents, Amersham Biosciences, Piscataway, NJ) and exposure to CL-XPosure film (Pierce Biotechnology, Rockford, IL) according to the manufacturer's instructions.

### GFP-HPS3 plasmid constructs

The *HPS3 *coding sequence was amplified from normal human cDNA [GenBank: NM_032383] with sequence specific primers and subcloned into pEGFP-C1 (Clontech, Palo Alto, CA). Site-directed mutagenesis to replace the clathrin-binding motif (LLDFE) at residues 172–176 with the non-conserved amino acids AAAPG was performed with the QuikChange Site-Directed Mutagenesis Kit (Stratagene, La Jolla, CA) according to the manufacturer's recommendations, using the forward primer 5'-AATGAGGAATTCTCAGCAGCGGCCCCTGGACGTTCTTTAATTATAC-3' and its reverse complement. All constructs were verified by sequencing before use.

### Transfections

All transfections were performed by electroporation in an Amaxa nucleofector electroporator (Amaxa GmbH, Germany). Electroporation of melanocytes was performed as described [42]. Fibroblasts were electroporated using Amaxa reagents and 3 μg of plasmid DNA with the U_23 nucleofector program.

### Fluorescence microscopy

Approximately 16 h after transfection with either GFP-HPS3 or GFP-HPS3-delCBD normal melanocytes were incubated at 20°C for 2 h to block trafficking out of the trans-Golgi [35,36]. Cells were transferred to 37°C for 5 min to release the temperature block and resume normal trafficking then fixed in 3% paraformaldehyde. Melanocytes allowed to express GFP-HPS3 or GFP-HPS3-delCBD for longer than 24 h demonstrated some GFP aggregates and decreased viability. Slides were blocked in PBS containing 0.1% saponin, 100 μM glycine, 0.1% BSA and 2% donkey serum followed by incubation with mouse monoclonal clathrin heavy chain antibodies (1:200 dilution; BD Biosciences Pharmingen). The cells were washed and incubated with donkey anti-mouse antibodies conjugated to ALEXA-555 (Molecular Probes), washed again, and mounted in VectaShield (Vector Laboratories, Burlingame, CA).

Live cell imaging was performed on HPS3 null fibroblasts expressing either GFP-HPS3 or GFP-HPS3-delCBD approximately 16 h after transfection. Cells were incubated at 20°C for 2 h followed by a 10 min incubation with the acidic vesicle dye Lysotracker Red (10 nM; Molecular Probes). The cells were then placed in fresh, pre-warmed culture media and imaged on a 37°C warming stage. All cells were imaged with a Zeiss 510 META confocal laser-scanning microscope (Carl Zeiss, Microimaging Inc., Germany) using a 488 Argon and a 543 HeNe laser. Images were acquired using either a Plan Apochromat 63X/1.4 oil DIC or a 100× Plan Apochromat 100X/1.4 oil DIC objective.

### Immunoelectron microscopy

Normal melanocytes were fixed approximately 9 h or 24 h after transfection in 2% paraformaldehyde with 0.2% glutaraldehyde in PHEM buffer for 2 h. After embedding, cutting, cryoprotection and snap-freezing of the pellet, ultrathin cryosections were labeled with mouse monoclonal antibodies to clathrin (1:100) (BD Biosciences Pharmingen). The mouse monoclonal antibodies were indirectly labeled with 20-nm protein A-gold particles via a rabbit anti-mouse IgG bridging antibody (1:200) (DakoCytomation, Denmark). The second labeling was performed with a rabbit polyclonal anti-GFP antibody (1:1000) [43], followed by 10-nm protein A-gold incubation. To exclude co-labeling artifacts, ultrathin cryosections were labeled with primary antibodies as above, but incubated with 10-nm protein A-gold in the first labeling and 15-nm protein A-gold in the second labeling. The grids were contrasted with uranyl acetate and imaged with a Philips EM 410 electron microscope (Philips, Eindhoven, The Netherlands). Quantitation of anti-GFP immunogold labeling of cellular compartments and its association with clathrin (anti-clathrin immunogold labeling) was performed on randomly selected cells expressing either GFP-HPS3 (28 cells) or GFP-HPS3-delCBD (25 cells) [44].

## List of abbreviations

HPS, Hermansky-Pudlak syndrome; BLOC, biogenesis of lysosome-related organelles complexes; GFP, green fluorescent protein; CBD, clathrin-binding domain; TGN, trans-Golgi network; DOPA, dihydroxyphenylalanine; PBS, phosphate buffered saline; BSA, bovine serum albumin; SDS-PAGE, sodium dodecyl sulfate-polyacrylamide gel electrophoresis; PHEM, PIPES, HEPES, EGTA, MgCl_2_

## Authors' contributions

AHW performed the immunoprecipitations. AHW and MH prepared the plasmid constructs. AHW and HD carried out the cell culture, transfection experiments, immunofluorescence and live cell confocal imaging. WAG recruited the HPS-3 patient and established the fibroblast cultures. RB established the HPS-3 patient melanocyte cultures. Immunoelectron microscopy and quantitation was performed by WW and MM. WAG, MH, WW and AHW prepared the manuscript.

## Supplementary Material

Additional file 1 **Time series of GFP-HPS3 and acidic vesicle live cell imaging**. GFP-HPS3 expressing HPS3 null fibroblast was imaged at 37°C following a 2 h incubation at 20°C. Time series (from inset in Figure 2a[A] and Figure 2b) showing a transient association of GFP-HPS3 with Lysotracker Red stained vesicles as they exit the perinuclear area. Click here for file
